# Simiate is an Actin binding protein involved in filopodia dynamics and arborization of neurons

**DOI:** 10.3389/fncel.2014.00099

**Published:** 2014-04-08

**Authors:** Kristin Derlig, Toni Ehrhardt, Andreas Gießl, Johann H. Brandstätter, Ralf Enz, Regina Dahlhaus

**Affiliations:** ^1^Department of Medicine, Emil-Fischer Centre, Institute for Biochemistry, University of Erlangen-NurembergErlangen, Germany; ^2^Department of Biology, Animal Physiology, University of Erlangen-NurembergErlangen, Germany

**Keywords:** dendritogenesis, filopodia, Simiate, Actin cytoskeleton, transcription

## Abstract

The Actin cytoskeleton constitutes the functional base for a multitude of cellular processes extending from motility and migration to cell mechanics and morphogenesis. The latter is particularly important to neuronal cells since the accurate functioning of the brain crucially depends on the correct arborization of neurons, a process that requires the formation of several dozens to hundreds of dendritic branches. Recently, a model was proposed where different transcription factors are detailed to distinct facets and phases of dendritogenesis and exert their function by acting on the Actin cytoskeleton, however, the proteins involved as well as the underlying molecular mechanisms are largely unknown. Here, we demonstrate that Simiate, a protein previously indicated to activate transcription, directly associates with both, G- and F-Actin and in doing so, affects Actin polymerization and Actin turnover in living cells. Imaging studies illustrate that Simiate particularly influences filopodia dynamics and specifically increases the branching of proximal, but not distal dendrites of developing neurons. The data suggests that Simiate functions as a direct molecular link between transcription regulation on one side, and dendritogenesis on the other, wherein Simiate serves to coordinate the development of proximal and distal dendrites by acting on the Actin cytoskeleton of filopodia and on transcription regulation, hence supporting the novel model.

## Introduction

The Actin cytoskeleton forms the functional base of a wide variety of cellular processes ranging from motility and migration to morphogenesis and structure maintenance, and even more versatile than the functions are, are the molecules regulating the assembly and disassembly of Actin filaments (for a comprehensive review of Actin binding proteins, pls. see Winder and Ayscough, 2005). Inside a cell, Actin can be found in two different forms, monomeric (globular) Actin denoted as G-Actin and polymeric (filamentous) Actin described as F-Actin. Since the polymerization of G-Actin is energetically discouraged until a trimer is formed, F-Actin assembly relies on monomer binding proteins that mediate the nucleation of a trimer, whereafter monomer delivering and polymerizing proteins together promote the filamentation at the barbed end, while monomer capping and sequestering proteins as well as barbed end capping proteins prevent F-Actin formation.

On the other side, the pointed end, F-Actin disassembly is expedited by severing and depolymerizing proteins, whereas proteins capping the filament impede the loss of Actin monomers from the pointed end. In doing so, Actin monomers actually released can be recycled: Following a nucleotide exchange to reconstitute the ATP bound form of G-Actin that is required for polymerization, the monomers can be used again to advance Actin polymerization at the barbed end, resulting in a directional filament propulsion. This process is described as Actin treadmilling.

The correct functioning of the Actin cytoskeleton requires further Actin binding proteins (ABPs), which orchestrate the Actin filaments into higher order structures such as Actin bundles, branches, and networks involving additional cellular components like membrane anchors, microtubules, intermediate filaments, or signaling cascades. Interestingly, some of these cytoskeleton regulators have been shown to use the conformational flexibility of Actin filaments to control the binding of other ABPs to the nearby filament (McGough et al., 1997; Chan et al., 2009), thereby providing not only a mechanism for the construction of different types of Actin networks in the same cellular compartment, but also to alter the properties of Actin filaments in a spatial manner. Indeed, in filopodia only two Actin bundling proteins have been found to be present along the entire length of the Actin filament (Svitkina et al., 2003; George et al., 2007, 2013), while most bundlers are localized to specific parts of the filopodium (Xue et al., 2010). In addition, it has been shown that Actin bundlers may quickly exchange between different filaments inside a filopodium (Nakagawa et al., 2006; Vignjevic et al., 2006), hence suggesting a model in which the localization and dynamic exchange of specific Actin bundling proteins control the motility or persistence of the filopodium.

By contrast, the formation and elongation of filopodia have been attributed to the filopodia head and the molecular machinery controlling Actin polymerization (Berg and Cheney, 2002; Svitkina et al., 2003; Lebrand et al., 2004; Yamagishi et al., 2004; Millard et al., 2005; Pellegrin and Mellor, 2005; Schirenbeck et al., 2006; Dent et al., 2007; Goh et al., 2011). Aside from the various functions of filopodia formation and extension in cellular activities such as environmental exploration or migration, the development of dendritic trees in neurons has recently been found to involve filopodia formation and subsequent stabilization as well (Niell et al., 2004; Hossain et al., 2012; Komaki et al., 2013). Live-imaging studies showed that fine filopodia emerge from growing dendrites, expanding for a period of ~20 min. and retracting again within about 1 h, but only some are stabilized and develop into mature, synapse holding dendrites (Niell et al., 2004; Hossain et al., 2012). Several factors have been proposed to be responsible for the stabilization of filopodia and their development into mature dendrites including extrinsic signals such as local calcium transients originating from other filopodia (Lohmann et al., 2002, 2005), neurotransmitter release (Rajan and Cline, 1998; Lohmann et al., 2002; Haas et al., 2006; Shen et al., 2009), cell adhesion molecules (Coppolino et al., 1997; McCroskery et al., 2006; Watson et al., 2007), and membrane tension (Heiman and Shaham, 2009), but also intrinsic determinants such as transcription factors have been shown to affect dendrite morphogenesis (reviewed in De La Torre-Ubieta and Bonni, 2011) and filopodia formation (Feng et al., 2013). However, the underlying molecular mechanisms are not well understood yet and in particular the proteins mediating those effects as well as the processes coordinating the different cellular machineries have remained elusive.

Here, we present a novel ABP, Simiate. Previous studies (Derlig et al., 2013) have revealed that Simiate is expressed in a wide variety of tissues and cell types and functions in transcription regulation. Now we show that Simiate directly associates with G- as well as F-Actin and localizes to dendrites and filopodia, where it is especially enriched in the tip. In doing so, Simiate is demonstrated to control Actin turnover, filopodia motility, and arborization of developing hippocampal neurons.

## Methods

### Biochemistry

#### Dyes

PhalloidinAx647 (F-Actin labeling; *Life Technologies*), DNaseIAx488 (G-Actin labeling; *Life Technologies*), DAPI (DNA-labeling; *Life Technologies*), Lifeact-RFP (Riedl et al., 2008) (F-Actin labeling during live imaging; *Ibidi*).

#### Antibodies

Primary antibodies: beta-Actin (mouse, *Abcam*; WB 1:5000; IHC 1:200), FAK (mouse, Abcam, IHC: 1:200), GFP (mouse; *Covance*; WB 1:2000), MAP2 (chicken, *Abcam*; IHC: 1:2500), Simiate [rabbit, (Derlig et al., 2013); WB: 1:2000; IHC: 1:200].

Secondary antibodies: HRP antibodies (*GE Healthcare*; WB 1:2000), Alexa-antibodies (*Life technologies*; IHC 1:500–1:1000), gtαmCy5 (*Abcam*; IHC 1:250).

#### Production of recombinant proteins

The cloning of Glutathione-sepharose-tag (GST)-Simiate as well as the purification of recombinant proteins have been described in detail previously (Derlig et al., 2013). In brief, GST or GST-Simiate were purified from *E. coli* BL21 Rosetta (*Novagen*) cells as outlined in the manufacturer's instructions (GST: *GE Healthcare*) using a french press (*Thermo Electron*) for lysis. 6His-Simiate (His: *Novagen*) was prepared correspondingly.

#### Coprecipitations

In order to identify potential interaction partners of Simiate, 100 μg GST-fusion proteins were covalently coupled to CNBr-activated sepharose 4 *(GE Healthcare)* according to the manufacturer's instructions and incubated with 2.5 mg mouse brain cytosol in Hepes-buffer (10 mM HEPES, pH 7.5; 1 mM EGTA; 0.1 mM MgCl_2_; 1% Triton; 150 mM NaCl). After three washing steps, proteins were eluted with 0.1 M Glycine, pH 2.6. Following neutralization using NaOH, the eluted proteins were precipitated with Trichlorine acetic acid, washed twice with Hepes-buffer and resolved in SDS-buffer. Proteins were then subjected to SDS-PAGE (28 cm, 7 mA, over night) and colloidal coomassie staining. Finally, bands of interest were excised and identified by MALDI-TOF analysis.

Alternatively, non-covalent coprecipitation assay were also employed. Therefore, Glutathione sepharose beads *(GE Healthcare)* carrying 100 μg of either GST-Simiate or GST-solo were processed as outlined above, but proteins were eluted at 95°C for 10 min in SDS-buffer (16% SDS; 40% Glycerine; 20% 2-Mercaptoethanol; 250 mM Tris-HCl, pH 6, 8; Bromophenol blue) prior to SDS-PAGE.

Non-covalent coprecipitations were also used to verify the association of Simiate and Actin. Therefore, Glutathione sepharose beads *(GE Healthcare)* carrying 30 μg of either GST-Simiate or GST solo were incubated over night at 4°C with 30 μg G-Actin (*Life Technologies*) in Hepes-buffer and processed as described above, before adsorbed proteins were eluted at 95°C for 10 min in SDS-buffer (16% SDS; 40% Glycerine; 250 mM Tris-HCl, pH 6, 8; Bromophenol blue) and subjected to SDS-PAGE and western blotting.

To demonstrate an interaction of endogenous Simiate and Actin, coimmunoprecipitations were performed. Following precipitation of endogenous Simiate from mouse brain cytosol (for details, please see Derlig et al., 2013), the coprecipitation of Actin was illustrated by western blotting.

#### Cosedimentation assay

G-Actin (*Life Technologies*) was polymerized for 2.5 h at 25°C in polymerization buffer (5 mM Tris pH 8.0, 0.2 mM CaCl_2_, 1mM ATP, 0.5 mM DTT, 5 mM KCl, 2 mM MgCl_2_) containing equal amounts (0.125 μg/μl) of either only G-Actin or G-Actin and recombinant Simiate, or a control protein (GST), respectively. Following ultracentrifugation at 100,000 g for 1 h to sedimentate F-Actin, the supernatant as well as the pellet were subjected to SDS-PAGE and western blotting to analyze the distribution of each protein by Ponceau staining or directly following SDS-PAGE by Coomassie staining, respectively. Alternatively, Actin was pre-polymerized, sedimentated, and subsequently incubated with Simiate or the control protein as outlined above. For further information on the interpretation, please also see the chapter “Bioinformatics and Statistics.”

### Histology

For details of the cultivation of human embryonic kidney (HEK-293) cells and primary hippocampal neurons as well as of immunhistochemistry experiments involving HEK-293 cells, neurons, and brain slices, please refer Derlig et al. (2013) and Dahlhaus et al. (2010). Live cell imaging was implemented using a laser scanning microscope (LSM 710, *Zeiss*) and ZEN 2010 software with corresponding imaging modules. The fluorescence recovery after photobleaching was implemented using consistent bleaching areas (size and relative location at the filopodia), while applying standard settings for all calculations.

### Bioinformatics and statistics

#### Morphometric analyses

Quantifications of dye intensities and morphometric measurements were carried out in ImageJ (*NIH*), while colocalization analyses were performed in ZEN2010 and Imaris (Bitplane). ImageJ was also employed to produce time – as well as z-projections from AVI-movies generated by ZEN2010 following live-imaging. Please note that the overlay of several images during projection results in a saturation of cellular structures. Using time-projections, the surveying activity of a cell was calculated as the total area covered in a given time utilizing migration, filopodia motility, and protrusion outgrowth minus the initial cell size. No credits are hence given for re-exploration of previously visited areas. Accordingly, the results are provided in μm^2^/h. The arborization of neurons was evaluated by Sholl analysis using the corresponding ImageJ plug-in with 14 concentrical cycles and a cycle distance of 100 μm.

#### Statistical testing

The statistical tests have been calculated in Prism (*GraphPad Software Inc.*) and Excel (*Microsoft Corp.*) as outlined in Derlig et al. (2013), except for the Welch's ANOVA and the subsequent Bonferroni *post-hoc* testing used to analyze the arborization of neurons, which were computed in MATLAB R2011. All other multiple *post-hoc* comparisons were reckoned according to Newman-Keuls (NK), since this test is less likely to give false negative results if several *post-hoc* comparisons are performed. For non-parametric testing of matched data, the Friedman test was applied along with Dunn's multiple comparison test (DMCT, Daniel, 1990). In all analyses, *p*-values are displayed by asterisks, while significance levels of *F*-tests are symbolized by clubs. Statistical values are reported in accordance with APA guidelines for statistical testing.

### Animal care

All animals were housed at the rodent facility of the Institute for Biochemistry according to the animal welfare conventions detailed recently (Derlig et al., 2013).

## Results

### Simiate and actin

**Figure 1:**
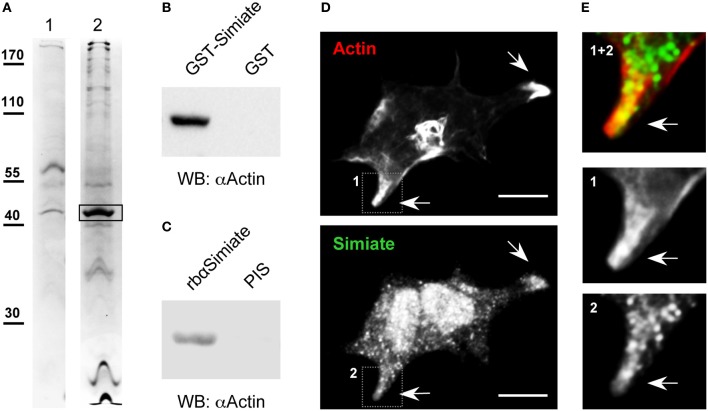
**Actin and Simiate associate. (A)** Representative acrylamide gel lanes from a coprecipitation assay with covalently coupled GST-Simiate and mouse brain cytosol. 1: GST control. 2: GST-Simiate. The box indicates a protein band subjected to MALDI-TOF analysis and identified as β-Actin in two independent experiments. **(B)** A coprecipitation assay with purified GST-Simiate and pure G-Actin, illustrated by western blotting with Actin-specific antibodies. **(C)** Coimmunoprecipitation of endogenous Simiate and Actin using Simiate-specific antibodies and mouse brain lysate. PIS: Preimmune serum. **(D)** Colocalization of Simiate and Actin. Actin is illustrated by expressing Liveact-RFP in HEK-293 cells, while Simiate was labeled by antibodies. The arrows indicate lamellipodia displaying a profound colocalization of Simiate and Actin. Scale bar: 10 μm. **(E)** Magnifications of the regions indicated in **(D)** (1 and 2).

Recently, we described a molecule named Simiate (Derlig et al., 2013). Simiate is an evolutionary old protein harking back to the origin of eukaryotes, which is expressed in a wide variety of tissues including heart, brain, liver, and kidney, and which localizes to nuclear speckles as well as to somata. Disabling the endogenous protein with specific antibodies demonstrated not only that Simiate functions in transcription regulation, but also that the protein is vital to cells. In order to learn more about the molecular mechanisms and cellular functions that Simiate takes part in we decided to search for its interaction partners. Therefore, we applied coprecipitation assays using GST-Simiate to accumulate its binding partners from mouse brain cytosol, subjected the samples to SDS-PAGE and identified bands of interest by MALDI-TOF analysis (Figure 1A). Employing GST for control, β-Actin was found to associate with Simiate in two independent coprecipitation experiments, a covalent and a non-covalent assay (Mascot scores: 95 and 90 with a significance level of *p* < *0.05* corresponding to a score of 70).

Next, we asked if the association of Simiate and Actin is direct or if other proteins are necessary to establish the protein complex. To address this question, a coprecipitation assay making use of purified GST-Simiate and straight G-Actin (Figure 1B) as the only proteins was conducted. Western blot analysis revealed that G-Actin specifically copurifies with Simiate. Hence, the interaction of Simiate and Actin is direct and requires no auxiliary proteins.

In order to evaluate whether endogenous Actin and Simiate also associate, coimmunoprecipitations were carried out (Figure 1C). While native Simiate was precipitated from mouse brain cytosol using specific antibodies covalently coupled to protein A agarose, coprecipitation of Actin was demonstrated by western blotting. This experiment illustrates that the two endogenous proteins associate as well.

An interaction of two proteins requires the presence of both binding partners at the same time at the same location inside a cell. Using Lifeact-RFP to label Actin and antibodies to stain Simiate (Figures 1D,E), a colocalization of both proteins was found in lamellipodia of HEK-293 cells (Figure 1E). As lamellipodia are characteristic morphological attributes of mobile cells, this finding suggests that the interaction of Actin and Simiate is relevant to cell morphology and/or cell migration.

**Figure 2:**
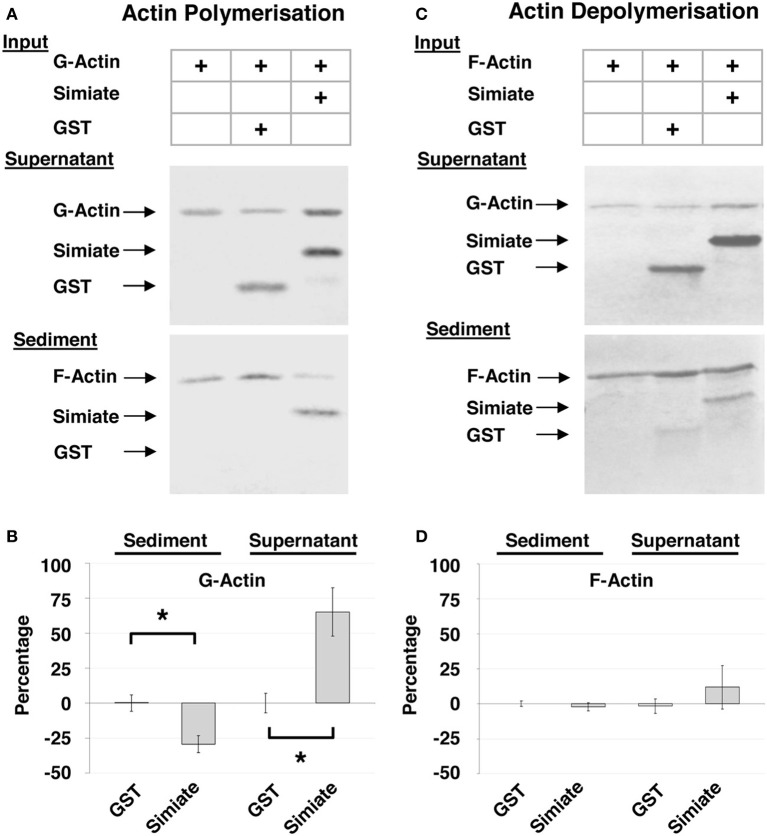
**Cosedimentation of Simiate and Actin. (A)** Cosedimentation assay with G-Actin. The pictures show a representative Ponceau staining out of five independent experiments. **(B)** Actin sedimentation. The graph illustrates the deviation from Actin control (Actin alone equals 0%) in percent and normalized to the total amount of Actin available (“Percentage”). **(C)** Cosedimentation Assay with F-Actin. The pictures show a representative Ponceau staining out of five independent experiments. **(D)** Same as **(B)**, but with F-Actin.

Within cells, Actin occurs as G-Actin and F-Actin and both types have specific as well as common binding partners, which regulate the polymerization and depolymerization of Actin in response to various signaling processes. Since our results have shown that Simiate directly associates with Actin, it is likely that Simiate also impinges on Actin polymerization or depolymerization. A cosedimentation assay availing ultracentrifugation to separate F- and G-Actin after a polymerization or depolymerization period was hence performed using affinity purified Simiate and GST for control (Figure 2).

The results illustrate that Simiate not only associates with G-Actin (cp. Figure 1C), but also directly binds to F-Actin (Figures 2A,C: Sediment; *51.4* ± *10% or 50.8* ± *8% of the total Simiate content*). In doing so, Simiate impairs the polymerization of G-Actin, while the unrelated control protein neither binds to F-Actin nor affects Actin polymerization (Figure 2A). A quantification (Figure 2B) revealed that the amount of Actin polymerized after a period of 3 h is reduced by 29% in the presence of Simiate [*H*_(*3*)_ = *25.92*, *p < 0.0001*; *post-hoc test (DMCT) p* < *0.05*], while the control protein has no significant influence (+0.25%) on the polymerization of Actin. Availing F-Actin instead of G-Actin in the sedimentation assay (Figures 2C,D), it turns out that Simiate again cosedimentates with F-Actin, but no depolymerizing effect is detected under the employed conditions. No influence of the stoichiometry was found either (data not shown).

### Simiate affects actin turnover and environmental exploration in living cells

**Figure 3:**
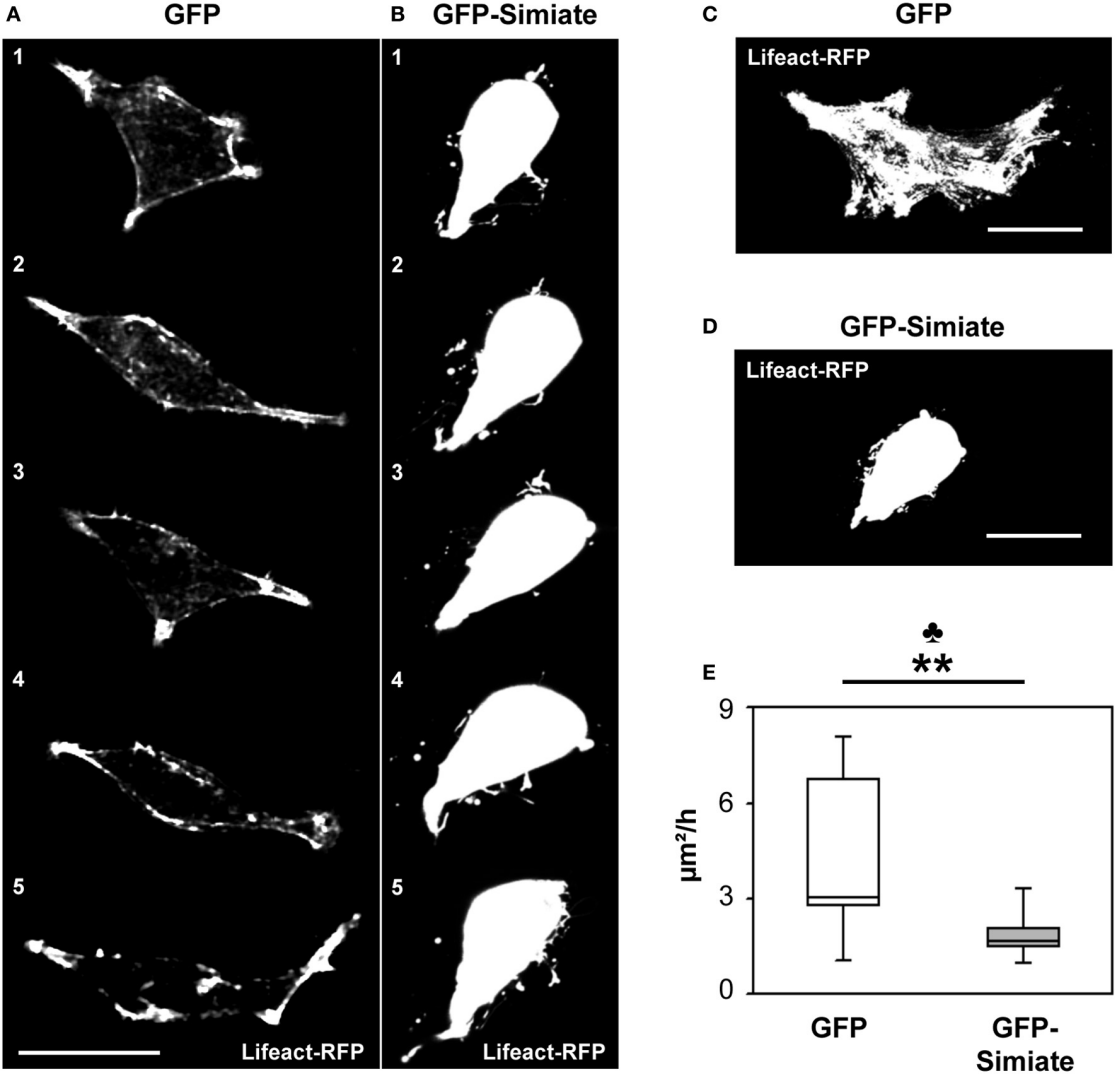
**Simiate influences the velocity of environmental exploration of cells. (A,B)** Time series images of the most active GFP **(A)** and GFP-Simiate **(B)** expressing HEK-293 cell (z-projections). Meantime between pictures (1–5): 24 min. **(C,D)** Time projections of a 2.2 h recording period from the same cells. Scale bar: 20 μm. Please note that the overlay of several images during projection results in a saturation of cellular structures. **(E)** Quantification of the areas explored. No credits were given for re-exploration of previously visited areas. The club indicates the significance level of the *F*-test, while the stars display the same for the *U*-test. GFP *n* = 12 and Simiate *n* = 11 cells. Average cell sizes: control −212 μm^2^, Simiate −204 μm^2^, difference insignificant.

Cell mobility is specifically depending on Actin turnover in lamellipodia and filopodia, with the latter serving to probe the environment during migration and to search for potential contacts. Indeed, our colocalization analysis (Figure 1D) suggests that the interaction of Simiate and Actin is most notably taking place in lamellipodia, hence implying an involvement in cell mobility. To test this hypothesis, we utilized HEK-293 cells as a model system and tracked cells with altered expression levels of Simiate over night. Since our previous experiments (Derlig et al., 2013) had shown that a decrease of functional Simiate is sufficient to induce massive apoptosis, we enhanced the availability of Simiate by expressing GFP-Simiate in our cells. Coexpression of Lifeact-RFP served to outline the morphology, while GFP was used as control. The novel area explored within 1 h was then calculated from the total area investigated by the cell (Figures 3A,B). Consequently, the measurements summarize all movements made by a cell to explore a novel environment, including migration, filopodia motility, and protrusion outgrowth. No difference was found in the exploration behavior of Lifeact-RFP or Lifeact-RFP and GFP expressing HEK-293 cells (data not shown).

By contrast, GFP-Simiate and GFP expressing cells exhibited clear distinctions in their surveying activity: While control transfected cells probed their environment with a exploration velocity of 3.1 μm^*2*^/*h* (*median* +*3.6 μ m*^*2*^/*h*, −*0.2 μ m*^*2*^/*h*, Figure 3C), GFP-Simiate expressing cells achieved only 1.7 μm^2^/h (*median* +*0.3 μ m*^*2*^*h*, −*0.2 μ m*^*2*^/*h*, Figure 3D), representing a highly significant reduction (*p < 0.01*, *U* = *20.00*). In addition, a significantly decreased range of variation (*F-test p* = *0.001*) was observed in GFP-Simiate expressing HEK-293 cells, suggesting that Simiate impairs the flexibility in the surveying activity of cells (Figure 3E).

Cell mobility and migration involve filopodia, which antennae-like explore the microenvironment in front of lamellipodia during cell motility. Such as in lamellipodia, filopodia mobility crucially depends on Actin dynamics. Given the impact of Simiate on cell motility and Actin polymerization, we hence hypothesized that Simiate may alter Actin dynamics in filopodia. To test this conjecture, we analyzed the fluorescence recovery after photobleaching (FRAP) of Lifeact-RFP labeled F-Actin in filopodia of GFP and GFP-Simiate transfected HEK-293 cells (Figure S1). In line with previous reports (reviewed in Mattila and Lappalainen, 2008) the results showed a range of half-times reaching from 4.13 to 28.47 s in GFP expressing cells (Figures S1A,C) and from 4.85 to 24.54 s in GFP-Simiate expressing cells (Figures S1B,D). Indeed, while there is no significant difference in the median F-Actin half-time of GFP and GFP-Simiate transfected cells (12.5 and 10.9 s), GFP-Simiate expressing cells experience a significantly reduced range of variation in their F-Actin half-times (*F-test p* = *0.0138*; Figure S1E). These findings illustrate that Simiate may impinge on cell motility and filopodia mobility by restricting Actin dynamics.

**Figure 4:**
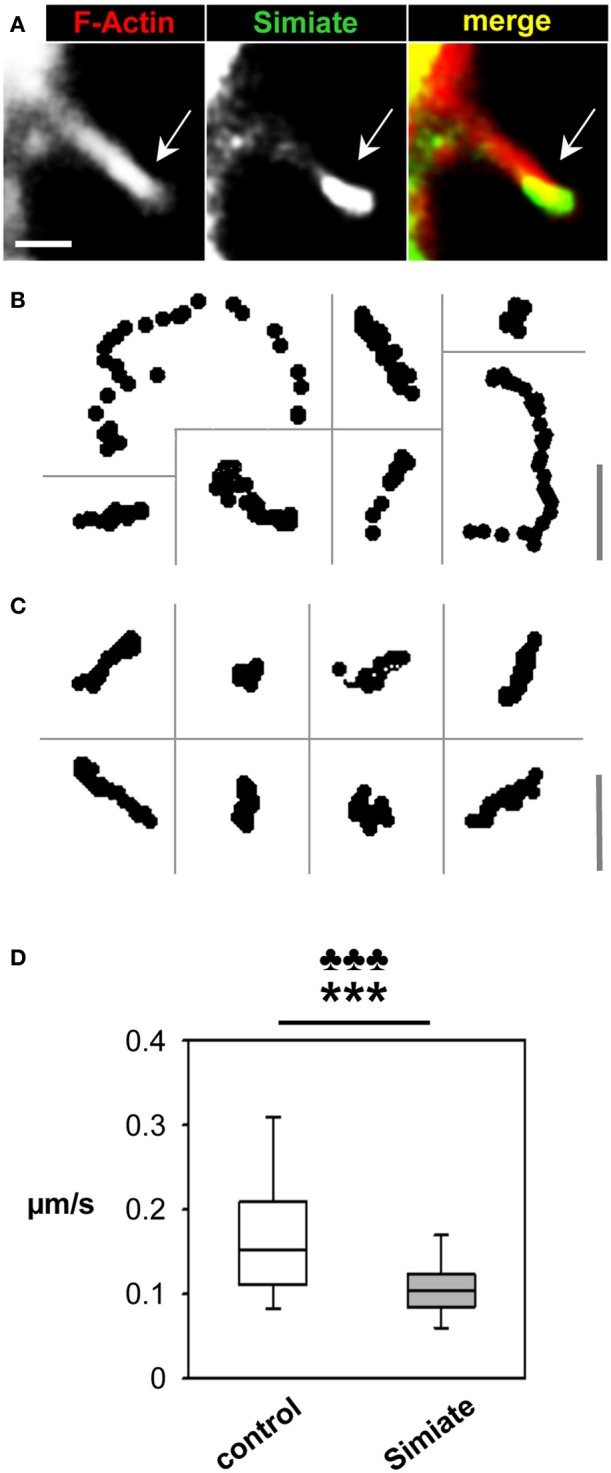
**Simiate affects filopodia dynamics. (A)** Colocalization of GFP-Actin and Lifeact-RFP labeled F-Actin in a filopodium protruding from a HEK-293 cell. Please note the accumulation of GFP-Simiate at the tip of the filopodium (arrow). In order to visualize the shaft of the filopodium as well, a picture with a higher gain is shown, resulting in signal saturation at the filopodia head. Scale bar: 2 μm. **(B)** Representative tracks of filopodia tips from GFP and Lifeact-RFP coexpressing HEK-293 cells. Scale bar: 3.5 μm. **(C)** Representative tracks of filopodia tips from GFP-Simiate and Lifeact-RFP coexpressing HEK-293 cells. Scale bar: 3.5 μm. **(D)** Quantification of filopodia head movements. The *p*-value of the *F*-test is represented by clubs, whereas the *p*-value of the *U*-test is shown as stars.

**Figure 5:**
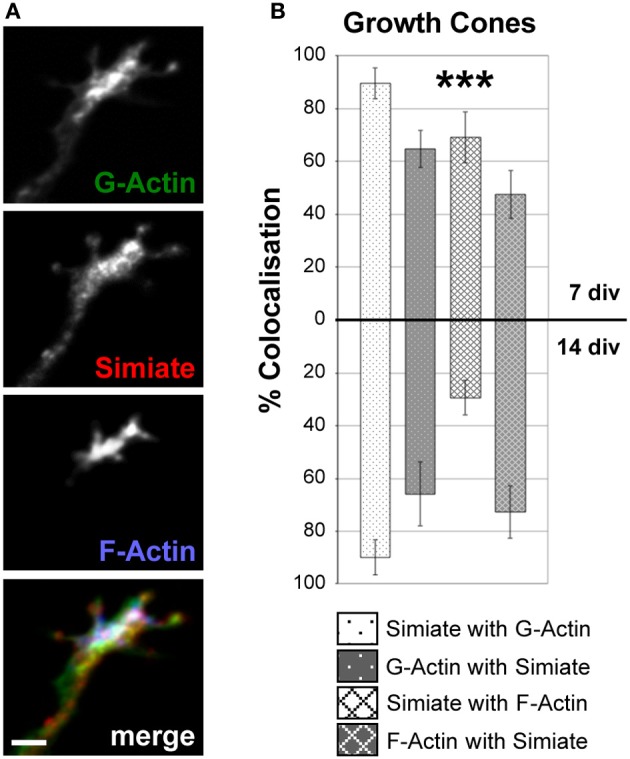
**Simiate colocalizes with G- and F-Actin in growth cones. (A)** An example of Simiate and G- as well as F-Actin colocalizing in a growth cone of a 7 div neuron from a low density culture of primary hippocampal cells. Scale bar: 2 μm. Endogenous F-Actin was labeled with Phalloidin, while DNaseI was used to identify G-Actin. **(B)** Quantification of the percental colocalization. Please note that growth cones were not differentiated into dendritic or axonal growth cones, but since dendritic growth cones are superior in numbers, most growth cones analyzed are dendritic.

In order to further evaluate the influence of Simiate on filopodia mobility, we analyzed the head movements of filopodia from GFP and GFP-Simiate expressing HEK-293 cells (Figure 4). Again, Lifeact-RFP served to visualize the cells and their filopodia. Interestingly, inside filopodia, GFP-Simiate localizes mainly to the head (Figure 4A) as does endogenous Simiate in filopodia from neuronal growth cones (cp. Figure 5A), supporting the idea that Simiate is indeed involved in the exploration behavior of filopodia by regulating Actin polymerization in the filopodia tip (cp. Berg and Cheney, 2002; Svitkina et al., 2003; Lebrand et al., 2004; Yamagishi et al., 2004; Millard et al., 2005; Pellegrin and Mellor, 2005; Schirenbeck et al., 2006; Dent et al., 2007; Goh et al., 2011).

Tracks recorded from filopodia heads (Figures 4B,C) indicate differences between GFP and GFP-Simiate expressing cells: While filopodia from GFP transfected cells show a diversity of track lengths within a given 30 s time frame, GFP-Simiate expression equalizes the track extensions. A quantification demonstrated that filopodia from GFP transfected cells display head movements with a median velocity of 0.15 μm/s (+*0.05 μ m/s*, −*0.04 μ m/s*), whereas filopodia of GFP-Simiate transfected cells manage only a median velocity of 0.11 μm/s (±*0.02 μ m/s*, Figure 4D). Both, the differences in the median and the variation, are highly significant (*F-test and U-test*: *p < 0.001*, *U* = *279.0*), hence suggesting that filopodia dynamics are particularly sensitive to the expression level of Simiate.

### Simiate and dendritogenesis

Given the direct association of Simiate with both, F- and G-Actin as well as its impact on Actin polymerization and filopodia dynamics on the one hand and the role of filopodia in contact formation and dendritogenesis on the other (Niell et al., 2004; Hossain et al., 2012; Komaki et al., 2013), we speculated that Simiate could be important to the organization of the Actin cytoskeleton in neurons, in particular during development. To address this hypothesis, we analyzed the colocalization of endogenous Simiate and G-Actin or F-Actin, respectively, in cultured hippocampal neurons of 7 and 14 div, the time when dendrites and synapses develop.

A prominent neuronal structure involved in contact formation and further demonstrating distinct Actin dynamics are growth cones, which are not only present at axonal tips, but also at the end of every single growing dendrite. Indeed, immunofluorescent colabeling of Simiate and F- as well as G-Actin illustrates that these proteins are enriched in growth cones (Figure 5A), where Simiate is also found at filopodia tips though it is absent from mature synaptic contacts (Derlig et al., 2013). Comparing growth cones from 7 and 14 div neurons, no major differences are seen except for a marked reduction in the colocalization of Simiate and F-Actin (Figure 5B). These observations suggest that Simiate functions specifically in dendritogenesis.

**Figure 6:**
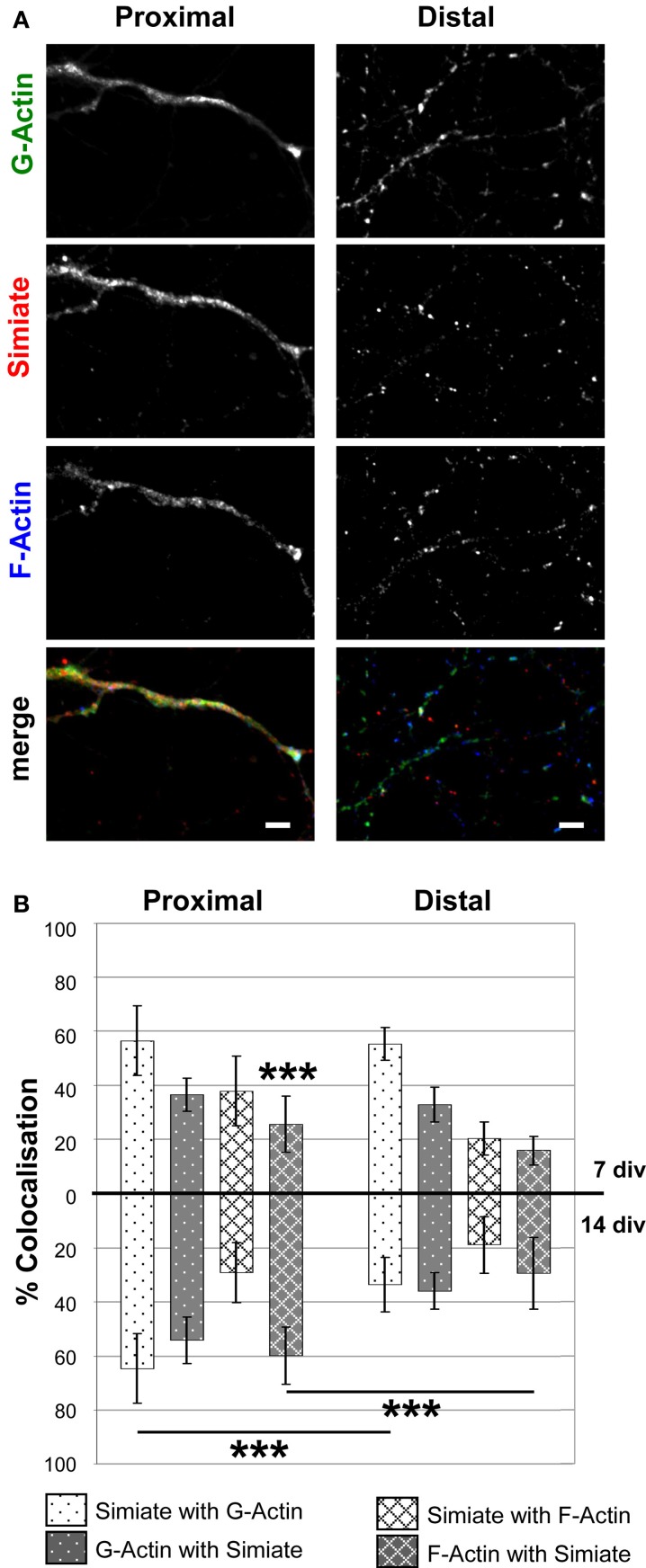
**Colocalization of Simiate with G- and F-Actin in proximal and distal dendrites. (A)** Simiate shows a higher colocalization with G- and F-Actin in proximal dendrites than in distal dendrites of 14 div neurons from primary hippocampal cells. Scale bar: 5 μm. Please see Figure 5 for further details. **(B)** Quantification of the percental colocalization as also shown in Figure 5B. Each of the eight 7 div colocalizations are significantly different compared to the respective 7 div data for growth cones shown in Figure 5B. For 14 div, this holds true only for the colocalization between Simiate with G-Actin. For the sake of clarity, significant differences between proximal or distal dendrites and growth cones are not graphically represented, but explained in detail in the results section.

Looking at proximal and distal dendrites (Figure 6), deviating colocalization patterns are noticed in 14 div neurons (Figures 6A,B), but not in 7 div neurons (Figure 6B). As no significant differences are seen in the amounts of Simiate, G- as well as F-Actin present at the specified locations when comparing 7 and 14 div neurons [*F _(1, 219)_* = *0.3014*, *p* = *0.5836*, *data not shown*], the alterations observed in 14 div neurons mainly reflect actual differences in the colocalization rather than altered expression levels. Hence, these findings indicate that the interaction of Simiate and Actin is relevant to the development of dendrites.

Interestingly, when comparing growth cones and dendrites, the colocalization of endogenous Simiate and Actin is regardless of the specific Actin species most evident in growth cones (cp. Figures 5A, 6A). This notion is supported by the quantification of the degree of colocalization between Simiate and F- or G-Actin (cp. Figures 5B, 6B). Here, 90% of Simiate colocalize with G-Actin in both, 7 and 14 div neurons, which is significantly more than in proximal [*F _(23, 220)_* = *20.54*, *p < 0.001*, *NK p < 0.001 for 7 div neurons and p* < *0.01 for 14 div neurons*] and distal dendrites (*NK p < 0.001 for both*, *7 div and 14 div neurons*). Further, in growth cones from 7 div neurons, 69% of Simiate also colocalize with F-Actin, which, again, is significantly more than in proximal (*NK p < 0.001*) and distal dendrites (*NK p < 0.001*), thus supporting the idea that Simiate is important to Actin dynamics.

Yet this relation is changed in growth cones of 14 div neurons, where only 29% of Simiate colocalize with F-Actin, which is significantly less than in 7 div neurons (*NK p < 0.001*) and indifferent from proximal or distal dendrites (*NK p > 0.05*). Interestingly, in 14 div neurons, there is also no difference in the percentage of G-Actin or F-Actin colocalizing with Simiate between growth cones and proximal dendrites, which, on the other hand, is the case in 7 div neurons (*NK p < 0.001*, *except for F-Actin and Simiate in growth cones vs. proximal dendrites: NK p < 0.05*). Hence, while there is a pool of Simiate associated G-Actin maintained in growth cones of 14 div neurons, the F-Actin pool changes, with the colocalization of Simiate and F-Actin as well as F-Actin and Simiate becoming more similar to the situation in proximal dendrites later in development. These findings suggest that the interaction of Simiate and Actin is differentially regulated in growth cones of 7 and 14 div neurons and that the properties of growth cones change during development. Further, since no significant differences are seen in proximal and distal dendrites from 7 div neurons, but from 14 div neurons (*NK p < 0.001 for Simiate and G-Actin as well as F-Actin and Simiate*), the results also imply that the interaction is important to the differentiation of dendrites and the arborization of neurons. Indeed, this idea is further encouraged by a significant increase in the percentage of F-Actin colocalizing with Simiate from 7 to 14 div neurons in proximal, but not distal dendrites (*NK p < 0.001*).

**Figure 7:**
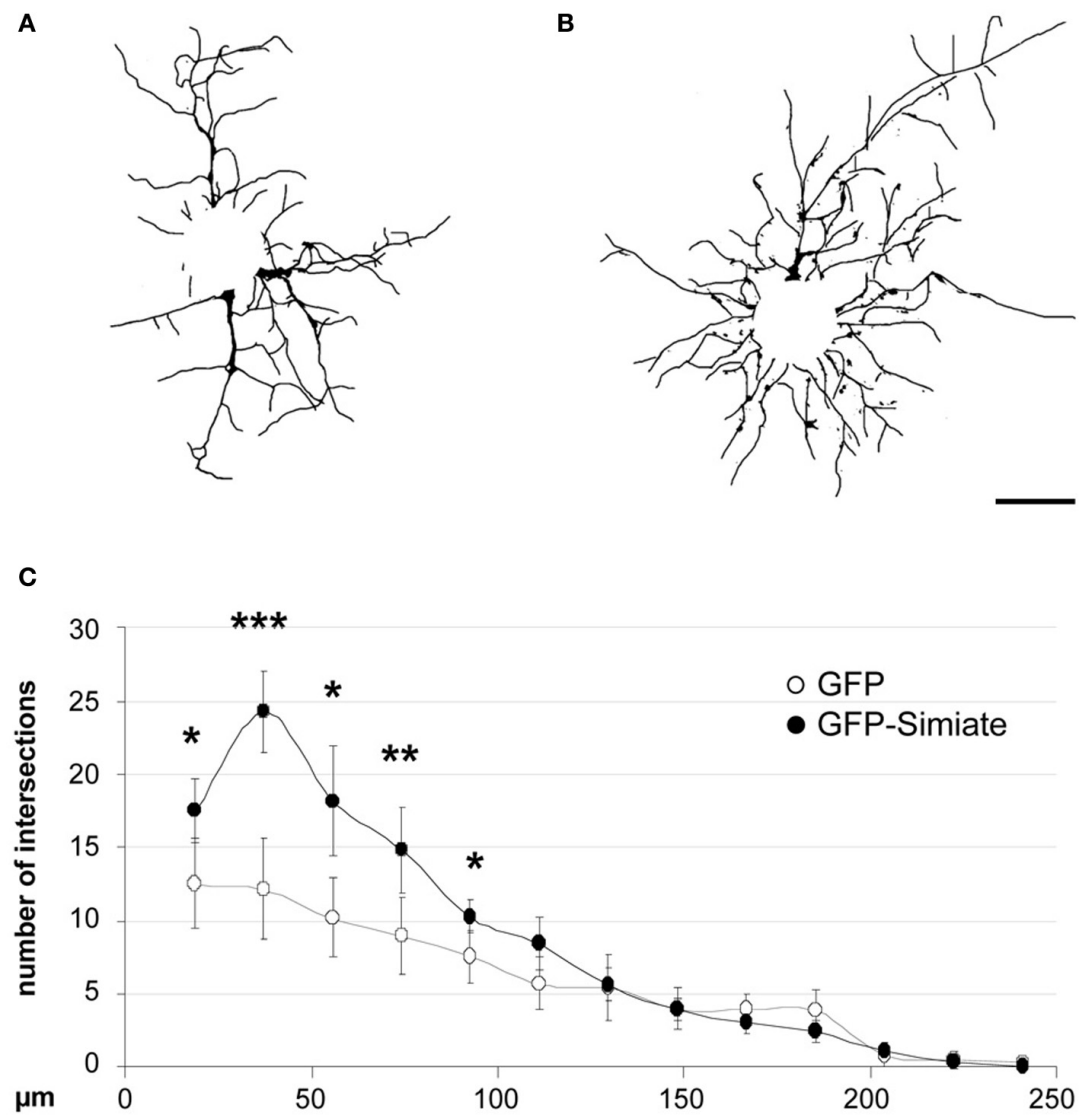
**Simiate is involved in the arborization of neurons. (A,B)** Reconstructed dendritic arborizations of representative pyramidal neurons expressing GFP **(A)** or GFP-Simiate **(B)**, respectively. Scale bar: 40 μm. **(C)** Sholl analysis comparing GFP (white circles) and GFP-Simiate (black circles) expressing pyramidal neurons of 7 div.

To test this hypothesis, we expressed GFP and GFP-Simiate for 24 h in developing hippocampal neurons and analyzed the arborization afterwards at 7 div by Sholl analysis (Figure 7). The results show that Simiate expressing neurons display a significant increase in the number of branches when compared to GFP expressing neurons [*F _(25, 92.5513)_* = *53.96*, *p < 0.001*]. A more detailed analysis revealed that the increase is highest around 36 μm, whereas no significant differences are observed beyond 100 μm, corresponding to alterations being present at the first 38% of the total dendrite length measured. No differences in the overall dendrite length are found. This experiment illustrates that Simiate specifically increases the branching in proximal dendrites.

**Figure 8:**
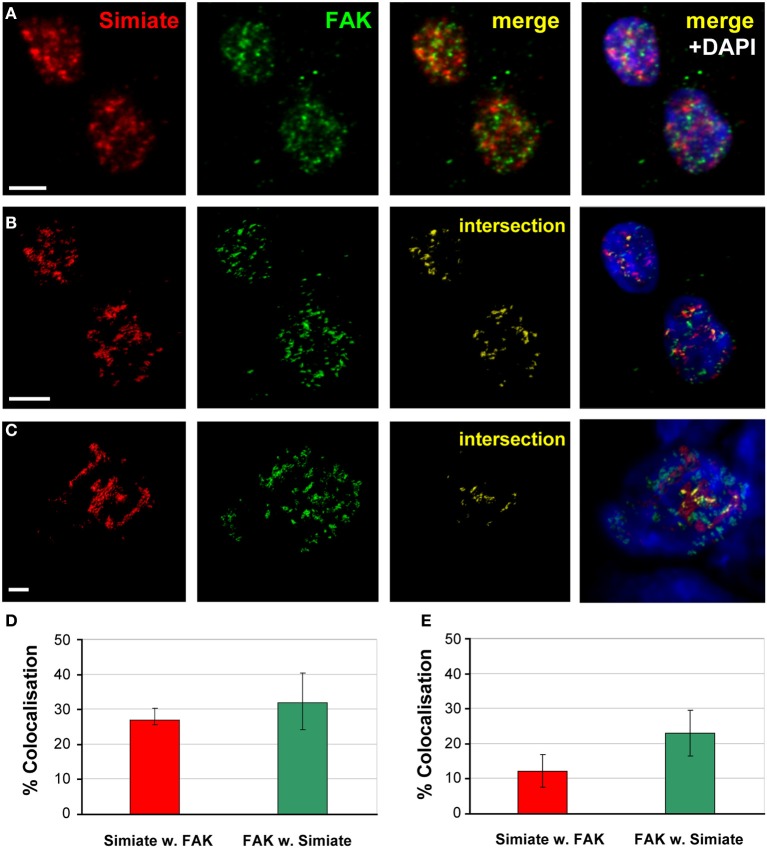
**Simiate and FAK colocalize in the nucleus. (A)** Z-Projections of stacks taken from immunofluorescent labeled HEK-293 cells. Scale bar: 5μm. **(B)** 3D-reconstruction of Simiate and FAK as shown in **(A)**. **(C)** 3D-reconstruction of Simiate and FAK in the nucleus of a pyramidal neuron from the CA1 region of the hippocampus. Scale bar: 2 μm. **(D,E)** Quantification of the colocalization of Simiate and FAK in HEK-293 cells (*n* = 17, **D**) as well as CA1 neurons (**E**, *n* = 14). Error bars indicate the confidence interval.

In this context, it is tempting to speculate that the effects Simiate exerts on cellular dynamics and the arborization of neurons, may involve interactions with focal adhesion sites. To address this question, we performed colocalization analyses in HEK-293 cells as well as in brain slices of 2.5 month old BL6 mice, using Focal Adhesion Kinase (FAK) specific antibodies to label corresponding sites (Figure 8). Strikingly, while neither in the somata and protrusions of HEK-293 cells nor in the Stratum radiatum of the Hippocampus any colocalization (≤*1.5 or 0.2%*, *respectively*) of Simiate and FAK was visible (data not shown), both proteins were found to colocalize in nuclei from HEK-293 cells (Figures 8A,B) and pyramidal neurons of the CA1 region (Figure 8C). A quantification of the degree of colocalization revealed that 27% of Simiate colocalize with FAK and 32% of FAK with Simiate in the nuclei of HEK293 cells (Figure 8D), while in the nuclei of CA1 neurons, 23% of FAK colocalize with Simiate and 12% of Simiate with FAK (Figure 8E). These findings imply that nuclear interactions involving Simiate and FAK are related to the function of Simiate in the organization of the Actin cytoskeleton.

## Discussion

Despite the progress made during the last decade in understanding the function of the Actin cytoskeleton in many cellular processes, its regulation in filopodia dynamics and dendritic development has not been well written on yet and especially the molecular mechanisms coordinating the different cellular machineries are still obscure. The data presented here now demonstrates that Simiate is an Actin binding protein, which not only regulates Actin polymerization and filopodia dynamics, but also represents a direct link to transcription modulation.

### Simiate directs actin polymerization

Unlike many other ABPs, Simiate is shown to bind to both, G-Actin and F-Actin. Though uncommon, some ABPs such as Profilins have been illustrated to interact with both isoforms (reviewed in Yarmola and Bubb, 2009), and just like Profilins, Simiate binding to G-Actin prevents spontaneous Actin polymerization, however, by contrast, the stoichiometry of Actin and Simiate did not influence the outcome. Interestingly, when F-Actin was offered to Simiate for binding, no depolymerizing effect of Simiate was seen, implying that Simiate *per se* is not able to depolymerize Actin filaments, but rather associates with Actin filaments laterally. Its influence on the polymerization of G-Actin further suggest that Simiate may also act as a G-Actin capping protein.

### The transcription factor—actin cytoskeleton model of dendritogenesis

Dendrite architecture has a significant impact on information processing in neurons (Spruston, 2008) and the variety of shapes observed in mammalian neurons suggests that a complex system of extrinsic and intrinsic mechanisms governs neuronal development. Recent studies (reviewed in De La Torre-Ubieta and Bonni, 2011) have given rise to a model where different transcription factors are detailed to distinct facets and phases of dendritogenesis, however, the proteins involved as well as the underlying molecular mechanisms are largely unknown. Interestingly though, components of the cytoskeleton such as microtubule binding proteins or Rho-GTPases, Gelsolin, and even Actin itself have been found to be targets of transcription factors in the context of dendritic arborization (Hand et al., 2005; Cobos et al., 2007; Wu et al., 2007; Li et al., 2010; Mokalled et al., 2010), suggesting that the dendritic development is governed by diverse connections of transcription factors on the one side and the cytoskeleton on the other. Indeed, our data further supports this model: Simiate not only modulates the Actin cytoskeleton by directly binding to G- and F-Actin and affecting Actin turnover, it also functions as a transcription modulator (Derlig et al., 2013) and alters dendritic branching specifically in proximal dendrites of hippocampal neurons. This result is supported by the observation that the colocalization of endogenous F-Actin and Simiate increases during dendritic development in proximal, but not distal dendrites of cultured neurons, hence not only implying that Simiate specifically acts on proximal dendrites, but also that the action involves an association of Simiate and F-Actin.

Notably, growth cones have been implicated in neuronal arborization as well (Hossain et al., 2012), since they are not only located at dendrite tips, but also serve in path finding by exploring the environment with their filopodia. The highest degree of colocalization between Simiate and Actin is indeed found in growth cones, where more than 80% of Simiate colocalize with G-Actin and more than 60% of G-Actin with Simiate. However, no change is observed during development, thus suggesting that the interaction of Simiate and G-Actin is not involved in neuronal arborization but rather elementary to growth cone function. By contrast, a significant decrease in the colocalization of Simiate and F-Actin is seen, hence implying that the behavior of growth cones changes during dendritogenesis and that these changes are modulated by the association of F-Actin and Simiate. These findings are in line with a recent study of growth cones conducted in *Xenopus laevis*, which illustrated by live-imaging that growth cone behavior varies with dendritic maturation, though the underlying molecular mechanisms remained unnamed (Hossain et al., 2012).

Strikingly though, the effect on Simiate and F-Actin seen in growth cones (decreased colocalization) is not only opposite to the effect observed in proximal dendrites (increased colocalization), but also mirrors the effects found by Sholl analysis: While there is a significantly elevated number of branches present at proximal dendrites, distal dendrites are unaffected, hence suggesting that Simiate stabilizes existing branches via its interaction with F-Actin following a rearrangement toward proximal dendrites. This idea is supported by our analysis of filopodia dynamics. Given the role of filopodia in dendritic arborization (Niell et al., 2004; Hossain et al., 2012; Komaki et al., 2013) and the couch potato behavior of filopodia under increased Simiate expression, it is likely that Simiate exerts its effects by regulating filopodia stability in dendrites and growth cones. In that Simiate works on growth cones *and* proximal dendrites, it may serve as a spatial coordinator during dendritogenesis (cp. Hossain et al., 2012).

Aside from its dendritic localization, Simiate is also present in the nucleus, where it strictly resides in nuclear speckles, small compartments that serve to organize the transcription and splicing machinery (Derlig et al., 2013). Applying specific antibodies to block the endogenous protein, we indicated Simiate to function in transcription regulation. Though these results suggest that the effects exerted by Simiate may include nuclear interactions, the effects Simiate exerts on cellular dynamics and the arborization of neurons could also be mediated by focal adhesion sites. Indeed, our experiments revealed that Simiate colocalizes with FAK, a marker for focal adhesions, however, not in the somata and protrusions of neuronal or HEK-293 cells, but in the nucleus. Sure enough, FAK has recently been found to function in transcription regulation by directing chromatin remodeling (Mei and Xiong, 2010; Schaller, 2010; Lim, 2013), hence supporting the idea that Simiate combines transcription and Actin dynamics to exert its effects and may even indirectly affect focal adhesion via interactions in the nucleus and/or via signaling cascades in the soma. Since the association of Actin and Simiate is in contrast to most of the above mentioned transcription factors, which impinge on the Actin cytoskeleton, direct and does not require any other proteins or signaling cascades, it is tempting to speculate that the mediation of these effects by Simiate may be comparable fast.

Taken together, our data suggests that Simiate functions as a direct molecular link between transcription regulation on one side, and dendritogenesis on the other, wherein Simiate serves to coordinate the behavior of growth cones with the development of proximal dendrites by acting on the Actin cytoskeleton of filopodia and the transcription machinery in the nucleus.

## Author contributions

Concept and design of the study: Regina Dahlhaus. Implementation of experiments: Regina Dahlhaus, Kristin Derlig, Toni Ehrhardt, Andreas Gießl. Data analysis: Regina Dahlhaus, Toni Ehrhardt, Kristin Derlig. Contribution of reagents/materials/tools: Andreas Gießl, Johann H. Brandstätter, Regina Dahlhaus, Ralf Enz. Writing: Regina Dahlhaus, Ralf Enz, Andreas Gießl, Johann H. Brandstätter, Kristin Derlig, Toni Ehrhardt.

### Conflict of interest statement

The authors declare that the research was conducted in the absence of any commercial or financial relationships that could be construed as a potential conflict of interest.

## Supplementary material

Figure S1 **Simiate influences Actin dynamics. (A–D)** Representative images illustrating fluorescence recovery after photobleaching (FRAP) in filopodia from Lifeact-RFP/GFP (“control”; **A,C**) and LifeactABD/GFP-Simiate (“Simiate”) transfected HEK-293 cells. **(A,C)** Control. **(B,D)** Simiate. Shown are the second slowest **(A,B)** and the second fastest **(C,D)** recovery observed (*n* = 22 per group), whereat gray dots indicate bleaching (at 3 s). **(E)** Quantification of the fluorescence recovery half-times after photobleaching in filopodia from GFP (“control”) and GFP-Simiate expressing HEK-293 cells.
