# Supplementary material for: Simiate is an Actin binding protein involved in filopodia dynamics and arborization of neurons
Source: Front Cell Neurosci. 2014 Apr 8;8:99. doi: 10.3389/fncel.2014.00099 (PMC3986562; doi:10.3389/fncel.2014.00099)
Supplement: Figure S1 — Simiate influences Actin dynamics. (A–D) Representative images illustrating fluorescence recovery after photobleaching (FRAP) in filopodia from Lifeact-RFP/GFP (“control”; A,C) and LifeactABD/GFP-Simiate (“Simiate”) transfected HEK-293 cells. (A,C) Control. (B,D) Simiate. Shown are the second slowest (A,B) and the second fastest (C,D) recovery observed (n = 22 per group), whereat gray dots indicate bleaching (at 3 s). (E) Quantification of the fluorescence recovery half-times after photobleaching in filopodia from GFP (“control”) and GFP-Simiate expressing HEK-293 cells. [file Presentation1.PDF]

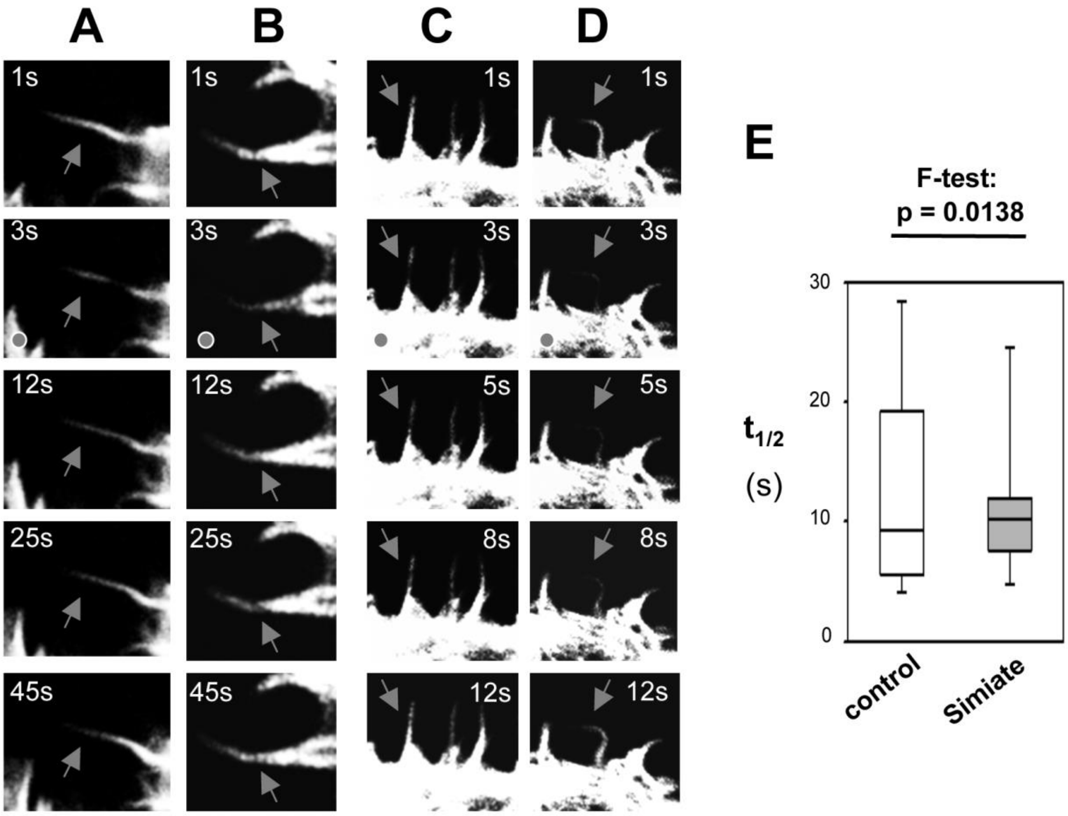

Figure S1: Simiate influences Actin dynamics. A-D) Representative images illustrating fluorescence recovery after photobleaching (FRAP) in filopodia from Lifeact-RFP/GFP ("control"; A, C) and LifeactABD/GFP-Simiate ("Simiate") transfected HEK-293 cells. A, C) Control. B, D) Simiate. Shown are the second slowest (A, B) and the second fastest (C, D) recovery observed (n=22 per group), whereat grey dots indicate bleaching (at 3s). E) Quantification of the fluorescence recovery half-times after photobleaching in filopodia from GFP ("control") and GFP-Simiate expressing HEK-293 cells.
